# Anatomic Characteristics of the Distal Oblique Bundle of the Interosseous Membrane of the Forearm

**DOI:** 10.7759/cureus.3964

**Published:** 2019-01-25

**Authors:** Stavros Angelis, Emmanouil Apergis, Georgios Vynichakis, Spyridon Triantafyllou, Panagiotis Skandalakis, Dimitrios Filippou

**Affiliations:** 1 Orthopaedics, General Hospital Hellenic Red Cross Korgialenio - Benakio, Athens, GRC; 2 Orthopaedics, General Hospital of Piraeus Tzaneio, Piraeus, GRC; 3 Urology, Medical School of National and Kapodistrian University of Athens, Athens, GRC; 4 Surgery, Medical School of National and Kapodistrian University of Athens, Athens, GRC

**Keywords:** distal oblique bundle, distal interosseous membrane, distal radioulnar joint

## Abstract

The distal oblique bundle of the forearm is a structure that has been under vigorous investigation for the past decade. It is part of the distal interosseous membrane (DIOM) and seems to have an important stabilizing effect in the distal radioulnar joint. In this essay, we have tried to summarize the anatomical characteristics of the structure. We have also compared and contrasted this to our own experience with eight freshly frozen forearms. It is our strong belief that the distal oblique bundle (DOB) may play a keystone role in future stabilization techniques of the distal radioulnar joint, and its anatomy characteristics need to be fully investigated.

## Introduction

Distal oblique bundle (DOB) is the thickest part of the distal interosseous membrane (DIOM) of the forearm. It has a very important stabilizing effect on the distal radioulnar joint [1-3]. It is known as a distinct structure since 2009, when Noda et al. first named it [1]. Their exact definition was as follows: “The DOB originated from approximately the distal one-sixth area of the ulnar shaft, approximately coinciding with the proximal border of the pronator quadrates muscle, and ran distally toward the distal radioulnar joint (DRUJ). The fibers blended into the capsular tissue of the DRUJ and eventually the DOB inserted to the inferior rim of the sigmoid notch of the radius. Furthermore, some fibers extended more distally along the anterior and posterior ridges of the sigmoid notch, so the DOB seemed to display continuity with the dorsal and palmar radioulnar ligaments of the triangular fibrocartilage complex (TFCC)”. This definition has been accepted in most of the relevant literature. In addition to this, Hohenberger et al. noticed one or more connecting fibers to the DRUJ or to components of the TFCC, in 85% of DOBs [4].

By reviewing the previous literature, it seems that Noda et al. were not the first to notice this structure. We noted that other authors may have stumped on the DOB, but never named it. For example, Gabl et al., in their figures, depicted the distal radioulnar tract, but they did not mention the bundle that courses volarly and opposite to it [5]. Moritomo mentioned later that the tract lies dorsally to the DOB and courses opposite to it [6-7]. Schneiderman et al., when describing the distal membranous portion of the interosseous membrane, mentioned some major bundles that course from the radius to the ulna and also the occasional appearance of one or two bands just proximal to the TFCC that course from the ulna to the radius [8]. Kapanji, in 1984, described a “tract” in the DIOM extending from the distal radius proximally to the ulna [9]. Earlier to this (1979), Kusswetter and Schmid had mentioned thin horizontal fibers between the distal radius and the ulna that inserted into the periosteum [10]. During our research, we also noted that way back in 1935, Lanz and Wachsmuth had described a structure that also coursed from the distal radius proximally to the ulna [11]. All these made us believe that the DOB is a structure of the DIOM that is known from the past. Probably, there are more references to it that are not known to the authors.

The aim of this study was to demonstrate the anatomic characteristics of the DOB, from our own experience with dissection of eight freshly frozen forearms, and also compare the results obtained in this study with the current literature.

## Materials and methods

Eight fresh-frozen forearms were dissected in our study. These samples came from one female and three male Caucasians. Hence, we had four right and four left hands four our disposal. The mean age was 61.5 years (range: 52-73 years). There were no signs of any pathology in our areas of interest.

After removal of all the soft tissues over the DIOM, the specimens were examined for the presence of a distinct DOB. Based on other studies, we described structures as DOBs only when their thickness was 0.5 mm or greater [4,6,12]. Measurements were taken with analog vernier calipers (KS Tools Pocket Vernier Calipers 0-150 mm 300.0510) with an accuracy of 0.05 mm.

**Figure 1:**
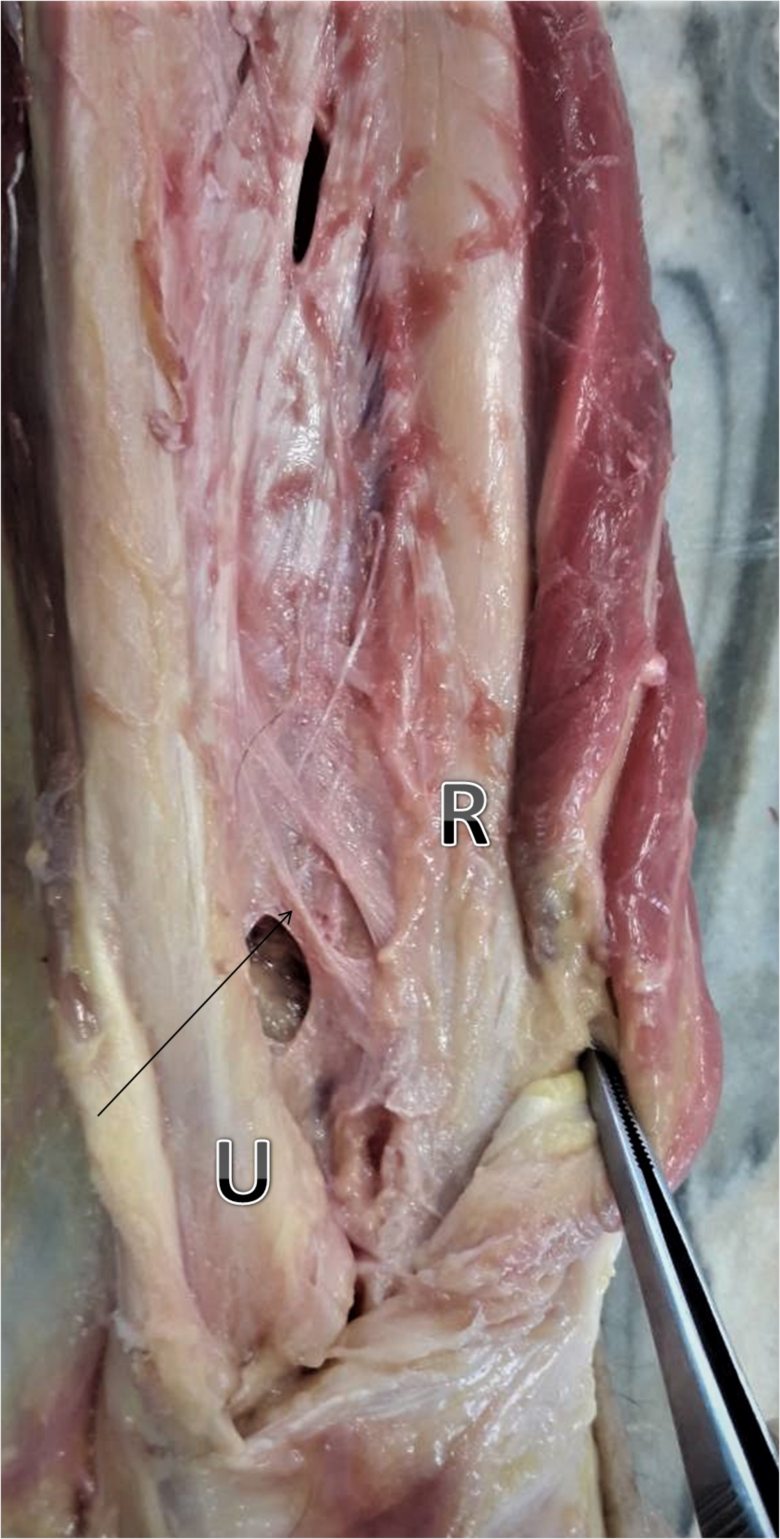
DOB (black arrow) is the thickest part of the DIOM. It originates from the distal one-sixth of the ulnar shaft and inserts the distal radius after an oblique course U: ulna, R: radius, DOB: distal oblique bundle, DIOM: distal interosseous membrane

If a DOB was present (Figure 1), we also measured its width and length and analyzed its points of insertion to the ulna and radius. Specifically, width was measured at the structure’s midpoint with the vernier calipers. The length was measured from the middle of the bundle’s attachment to the ulna to the middle of the bundle’s attachment to the radius. The distance from the middle of the bundle’s insertion to the ulna to the tip of the styloid process of the ulna as well as the distance from the middle of its insertion to the radius to the tip of the radiuses’ styloid process were calculated. 

## Results

**Figure 2:**
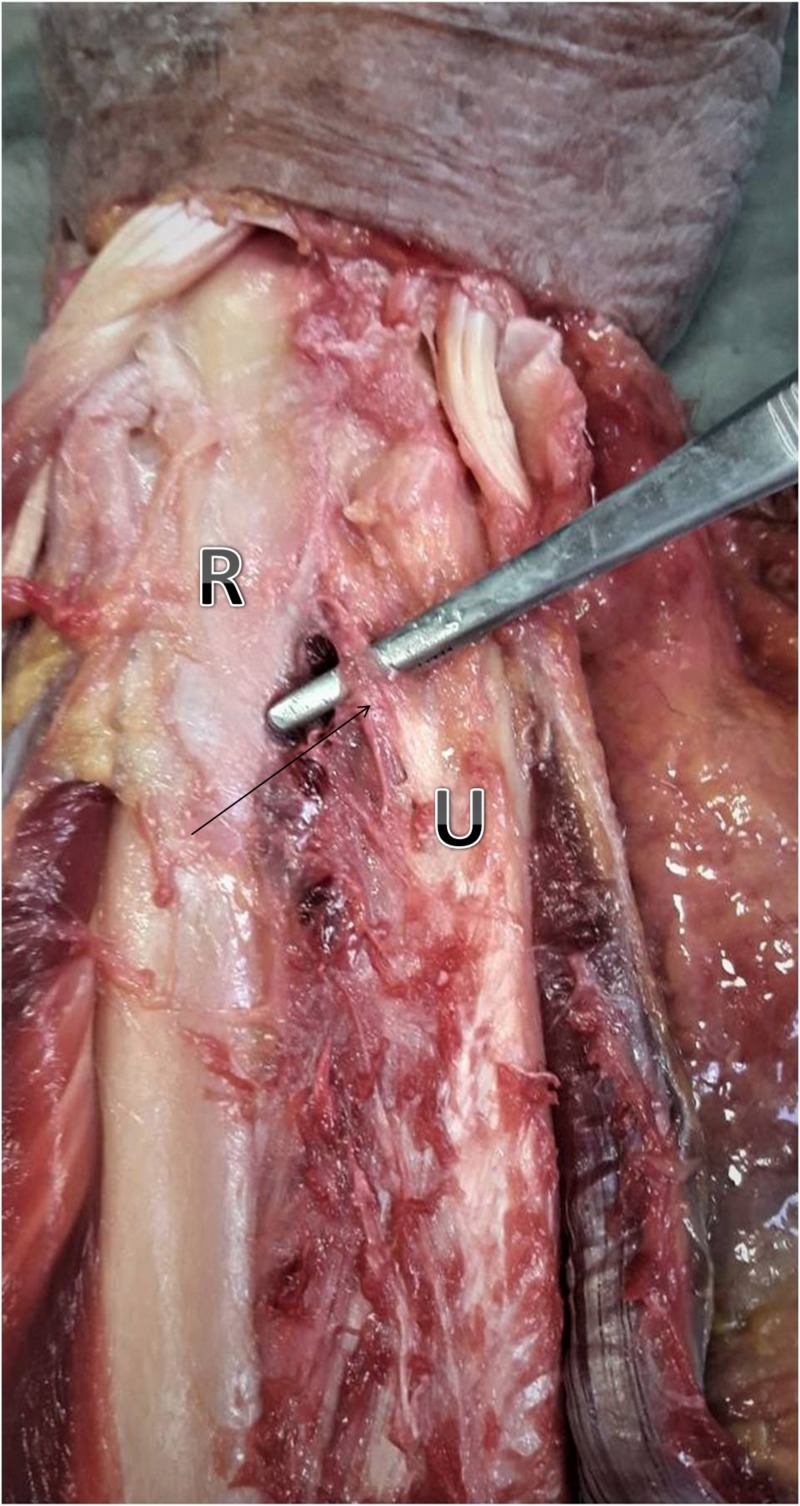
Distal oblique bundle (black arrow) of a freshly frozen female specimen U: ulna; R: radius

In our study of eight freshly frozen forearms, we found three DOBs, and, the prevalence was 37.5%. Two were found on men’s right hands and one on a female’s left hand (Figure 2).

The mean thickness was 0.83 mm (range: 0.6 to 1.0 mm). The mean width was 4.2 mm (range: 2.2 to 6.2 mm) and mean length 25.4 mm (range: 23.2 to 27.5 mm). Proximally, the mean distance from the middle of the bundle’s ulnar insertion to the tip of the styloid process of the ulna was 50.3 mm (range: 45.5 to 53.4 mm). Distally, the mean distance from the middle of the bundle’s insertion to the radius to the tip of the styloid process of the radius was 34.0 mm (range: 31.3 to 37.7 mm). Table 1 summarizes all these measurements.

**Table 1:** Summary of our measurements (mm) *: Distance from the tip of the ulnar styloid process, **: Distance from the tip of the radial styloid process

DOBs found	1 (right-male)	2 (right-male)	3 (left-female)
Thickness	0.9	1.0	0.6
Width	4.3	6.2	2.2
Length	25.6	27.5	23.2
Ulnar insertion*	53.4	51.9	45.5
Radial insertion**	37.7	31.3	33.1

## Discussion

In our study, we have dissected eight freshly frozen forearms in order to make measurements around the distal oblique bundle. We have compared these findings to the rest of the literature on the topic.

First, there seems to be some dispute around the prevalence of the DOB in the forearm. We report 37.5%, but our study sample is very small. In our opinion, Hohenberger et al.’s work on 185 forearms [4] is most relevant in this regard. They reported a prevalence of 29 % (53 specimens out of the 185 had a DOB), while smaller samples of about 10 to 30 forearms reported a prevalence of 40% [1-2,12]. Dy et al. reported a prevalence of 50% [13]. Of course, we should mention that Hohenberger et al. used cadaveric and not freshly frozen hands. This could play a role in the measurement of the thickness due to the probable loss of volume during the process of embalming. To our knowledge, only one study has reported prevalence results similar to those reported by Hohenberger et al. Kim et al. used MRI measurements in 80 specimens and calculated prevalence of 32.5% [14]. In their paper, however, structures are named DOBs if they have a thickness over 1.0 mm and not 0.5 mm. This means that if they had used 0.5 mm as their reference point, their prevalence results would probably be much higher.

In our study, the mean thickness of the bundle was 0.83 mm (range: 0.6 to 1.0 mm). This is comparable to the measurements of Hohenberger et al. [4], who report 0.9 mm (range: 0.5 to 1.8 mm) and Dy et al. [13], who report 0.85 mm ± 0.28 mm (range: 0.64 to 1.33 mm). Other authors reported thicker DOBs. Okada et al., in his ultrasound research of 14 forearms [15], estimated the mean thickness of the bundle to be 1.09 mm. Moritomo and Kitamura et al., respectively, reported 1.2 mm (range: 1.0-1.3 mm) [6,12]. The largest measurements were made by Noda et al. [1]. They reported 1.5 mm ± 0.5 mm (range: 0.5 to 2.6 mm). Kim et al., who used 1.0 mm as the reference, found the mean thickness to be 1.4 mm (range: 1.1 to 1.7 mm) in the DOB group and 0.6 mm (range: 0.2 to 0.9 mm) in the non-DOB group [14].

Kitamura et al. stated that the bundle may have some variations in its morphology [12]. They described some of those variations. In our sample, all DOBs were linear and thick structures that extended between the ulna and the radius at the course described by Noda et al. [1]. Hence, our width measurements were easy to perform in the middle of the structure. We reported a mean width of 4.2 mm (range: 2.2 to 6.2 mm). In these measurements, we have noticed a high range between the widths of our specimens. The same observation was made by Hohenberger et al. [4], who reported a mean width of 9 mm (range: 4 to 19 mm) and also by Noda et al., who reported 4.4 mm ± 1.1 mm (range: 2 to 6 mm) [1].

As far as length is concerned, there seems to be an agreement between the authors (~24 to 26 mm). We reported a mean length of 25.4 mm (range: 23.2 to 27.5 mm). Moritomo et al. reported a mean length of 26 mm ± 3.4 mm [2], Omori et al. 25.7 mm [16], and Hohenberger et al. 24 mm (range: 19 to 30 mm) [4].

There also seems to be a consensus between the authors about the points of insertion of the bundle in the ulna and the radius. We found that the mean distance from the middle of the bundle’s ulna insertion to the tip of the styloid process of the ulna was 50.3 mm (range: 45.5 to 53.4 mm). This is comparable to Kitamura et al.’s calculation of the distance of the proximal border of the DOB to the ulna from the head of the ulna, that is, 54 mm (range: 50 to 57 mm) [12]. We also agree with Hohenberger et al.’s mean distance of the proximal border of the DOB’s attachment to the ulna from the styloid process of the ulna, which is to be 48 mm (range: 34 to 65 mm) [4]. Noda et al. reported this distance to reflect on the 15% ± 2% (range: 13% to 21%) of the ulna’s length [1].

In our study, the mean distance from the middle of the bundle’s insertion to the radius to the tip of the styloid process of the radius was 34.0 mm (range: 31.3 to 37.7 mm). This agrees with Kitamura et al. who reported a mean distance of 35 mm between the proximal border of the DOB’s insertion to the radius and the styloid process of the radius [12]. Hohenberger et al. also reported 35 mm (range: 24-53 mm) [4], and Noda et al. found this distance to reflect on the 9.9 % ± 0.8 % (range: 8.3% to 11%) of the radius length [1].

All these measurements are important and seem to have a huge impact on surgical reconstruction procedures that have timidly appeared in international literature [17-20]. These procedures seem to be a less invasive promising alternative for the stabilization of the DRUJ.

Our study has some limitations. The major one is that our sample is very small and so may not have much impact on the literature. On the other hand, comparing our results to other authors has helped us draw useful conclusions.

## Conclusions

There seems to be a consensus between authors on the matters of the measurements of the bundle's length and points of insertion in the ulna and radius. On the other hand, there seems to be a difference between the prevalence and thickness of the structure. In our opinion, all these measurements are important and need to be performed on a larger scale and in a more categorized manner for more objective results and conclusions. After only 10 years of research, reports of simple reconstructions of the distal oblique bundle seem to be a very promising alternative for the stabilization of the DRUJ. Knowing now that the DOB acts as an isometric stabilizer of the DRUJ makes us support its full investigation.
